# PKA inhibition kills l-asparaginase-resistant leukemic cells from relapsed acute lymphoblastic leukemia patients

**DOI:** 10.1038/s41420-024-02028-w

**Published:** 2024-05-27

**Authors:** Jung Kwon Lee, Xidi Wang, Jinghua Wang, Jesusa L. Rosales, Ki-Young Lee

**Affiliations:** 1 Department of Cell Biology & Anatomy, Arnie Charbonneau Cancer and Alberta Children’s Hospital Research Institutes, University of Calgary, Calgary, AB Canada; 2 Department of Pathogen Biology and Immunology, Health Science Center, Ningbo University, Ningbo, China; 3 Department of Hematology, Second Affiliated Hospital, Harbin Medical University, Harbin, China

**Keywords:** Apoptosis, Leukaemia

## Abstract

Despite the success in treating newly diagnosed pediatric acute lymphoblastic leukemia (aLL), the long-term cure rate for the 20% of children who relapse is poor, making relapsed aLL the primary cause of cancer death in children. By unbiased genome-wide retroviral RNAi screening and knockdown studies, we previously discovered opioid receptor mu 1 (OPRM1) as a new aLL cell resistance biomarker for the aLL chemotherapeutic drug, l-asparaginase, i.e., OPRM1 loss triggers l-asparaginase resistance. Indeed, aLL cell OPRM1 level is inversely proportional to l-asparaginase IC50: the lower the OPRM1 level, the higher the l-asparaginase IC50, indicating that aLL cells expressing reduced OPRM1 levels show resistance to l-asparaginase. In the current study, we utilized OPRM1-expressing and -knockdown aLL cells as well as relapsed patient aLL cells to identify candidate targeted therapy for l-asparaginase-resistant aLL. In OPRM1-expressing cells, l-asparaginase induces apoptosis via a cascade of events that include OPRM1-mediated decline in [cAMP]_i_, downregulation of PKA-mediated BAD S_118_ phosphorylation that can be reversed by 8-CPT-cAMP, cyt C release from the mitochondria, and subsequent caspase activation and PARP1 cleavage. The critical role of PKA inhibition due to a decrease in [cAMP]_i_ in this apoptotic process is evident in the killing of OPRM1-knockdown and low OPRM1-expressing relapsed patient aLL cells by the PKA inhibitors, H89 and 14–22 amide. These findings demonstrate for the first time that PKA can be targeted to kill aLL cells resistant to l-asparaginase due to OPRM1 loss, and that H89 and 14–22 amide may be utilized to destroy l-asparaginase-resistant patient aLL cells.

## Introduction

With increased intensity in chemotherapy for acute lymphoblastic leukemia (aLL), the long-term survival in newly diagnosed diseases, especially in children, has amazingly improved. However, a subgroup of patients acquires chemotherapy resistance, and relapsed/refractory disease in children and adults have a 5-year survival estimate of ∼20% [1] and <10% [2], respectively. For example, patients treated with the strategic chemotherapy drug, l-asparaginase, show greater remission induction compared to those untreated with l-asparaginase, but while l-asparaginase clearly benefits a significant subset of aLL patients, some patients relapse due to the development of drug resistance. Thus, efforts have been directed at the pursuit of novel targeted therapies for patients that have developed resistance to key aLL chemotherapy drugs such as l-asparaginase.

Recently, we reported opioid receptor mu 1 (OPRM1) as a new biomarker for l-asparaginase resistance in aLL cells [3]. OPRM1 was identified through RNAi screening and knockdown studies. Analysis for the clinical relevance of OPRM1 as an aLL cell biomarker for l-asparaginase resistance was performed in aLL patient leukemic cells, which revealed that cells expressing relatively reduced levels of OPRM1 exhibit resistance to l-asparaginase while those expressing higher levels of OPRM1 are sensitive to l-asparaginase treatment. This indicates that presence or absence of OPRM1 governs the fate of aLL cells following treatment with l-asparaginase: i.e., gain causes cell death while loss causes survival or resistance. Altogether, these findings indicate that OPRM1 loss has a critical role in the development of resistance to l-asparaginase in aLL patients and that OPRM1 is essential for l-asparaginase to cause aLL cell death. However, the molecular mechanism through which l-asparaginase causes aLL cell death via the OPRM1 pathway is still unknown.

OPRM1, which is also known as mu opioid receptor 1 (MOR1), is known as the principal site of target of the commonly used opioid drugs such as morphine, heroin, fentanyl and methadone. It also acts as the target receptor for the endogenous opioids, beta-endorphin and enkephalins. OPRM1, a G-protein-coupled receptor, stimulates the G_i_ alpha subunit, negatively regulating the activity of adenylate cyclase that produces cyclic adenosine 3′,5′-monophosphate (cAMP) from ATP, thus reducing intracellular cAMP level ([cAMP]_i_). cAMP is an intracellular second messenger that serves a function in a number of biological processes, including apoptosis [4]. In fact, stimulation of OPRM1 by methadone was shown to reduce [cAMP]_i_, resulting in the activation of caspases and inducing apoptosis in leukemic cells [5], but how reduced [cAMP]_i_ causes caspase activation remains to be determined.

The intrinsic apoptotic pathway is regulated by the mitochondria through modulation of mitochondrial outer membrane permeabilization (MOMP). MOMP, in turn, is controlled by interactions between the anti-apoptotic (e.g., BCL-2) and pro-apoptotic (e.g., BAX, BAK, BAD) members of the BCL-2 family of proteins. Interactions are dependent upon their relative abundance and affinities [6], but phosphorylation of BAD (e.g., at S_118_) through cAMP-dependent protein kinase A (PKA) activity also triggers dissociation of anti-apoptotic BCL-2 from its neutralized form, BCL-2–BAD complex [7, 8]. Thus, PKA phosphorylation of BAD activates the anti-apoptotic role of BCL-2, negatively regulating MOMP and inhibiting the release of mitochondrial intermembrane space proteins, consequently inhibiting apoptosis as well. On the other hand, reduced [cAMP]_i_ and corresponding loss of PKA activation promote the formation of the BCL-2–BAD complex, which counteracts the anti-apoptotic activity of BCL-2, allowing BAK and BAX to create pores in the outer mitochondrial membrane, and causing cytochrome C (cyt C) release, caspase activation, and apoptosis [9, 10].

Using continuously growing OPRM1-expressing and -knockdown aLL cells as well as aLL cells isolated from relapsed patients, we demonstrate in the current study that l-asparaginase causes an OPRM1-mediated decline in [cAMP]_i_ that is linked to reduced PKA-associated phosphorylation of BAD at S_118_, increased cyt C release, caspase activation, and aLL cell apoptosis. Notably, we demonstrate that the PKA inhibitors, H89 and 14–22 amide, kill l-asparaginase-resistant aLL cells depleted of OPRM1 as well as l-asparaginase-resistant patient aLL cells expressing reduced levels of OPRM1.

## Results

### l-asparaginase induces aLL cell apoptosis via the OPRM1 pathway that leads to a decline in [cAMP]_i_

**Fig. 1:**
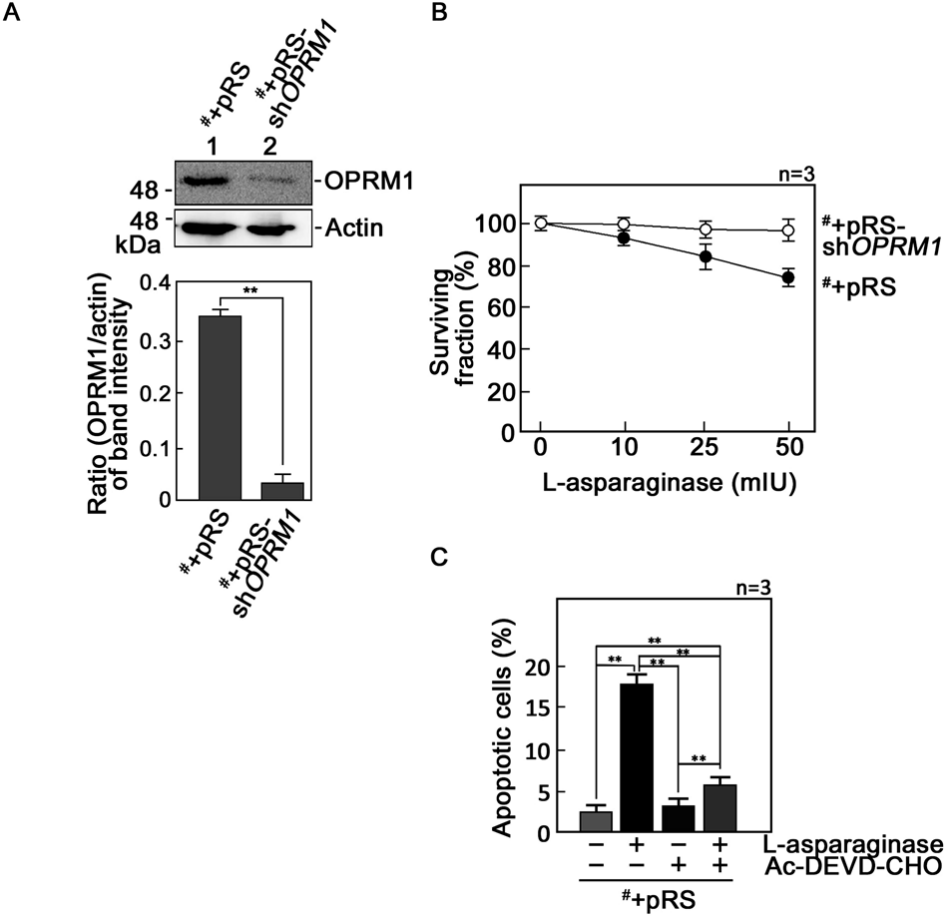
Loss of OPRM1 confers resistance to l-asparaginase. **A** Knocking down OPRM1 in parental POETIC2 cells (^#^) by infection with retrovirus carrying pRS-sh*OPRM1* (^#^+pRS-sh*OPRM1*). Lysates (40 μgs) of ^#^+pRS-sh*OPRM1* cells and cells infected with an empty pRS vector (^#^+pRS) were subjected to SDS–PAGE and immunoblotting for ORPM1 (upper panel) and actin (loading control, lower panel). Representative blots (upper panel) from one of three independent experiments (*n* = 3) showing similar results are shown. The bottom panel shows the ratios of OPRM1 levels vs actin levels based on densitometric analysis of the blots using the NIH ImageJ 1.61 software. Actin values were normalized to 1.0. Calculated ratios are means ± SD of the three independent experiments. **p < 0.05. **B** ^#^+pRS (●) cells show decreasing viability (i.e., increasing sensitivity) to increasing concentrations of l-asparaginase while ^#^+pRS-sh*OPRM1* cells (○) exhibit resistance to l-asparaginase. Cells were treated with l-asparaginase for 1 day and cell viability was quantified using Alamar blue assay. Values are means ± SEM from three independent experiments. **C** ^#^+pRS cells pre-treated (or not pre-treated) with 50 µM Ac-DEVD-CHO (a caspase inhibitor) for 3 h then treated or untreated with 50 mIU/ml l-asparaginase for 16 h were double-stained with propidum iodide (PI)- and FITC-labeled Annexin V. FITC-positive apoptotic cells were counted at ×10 magnification using a I × 71 Olympus inverted microscope attached to a 37 °C incubator with 5% CO_2_. The percentage of FITC-positive apoptotic cells was determined from a field of ∼100 PI-stained cells using the Olympus CellSens software (Olympus, Japan). Values are means ± SD of the three independent experiments. **p < 0.05.

**Fig. 2:**
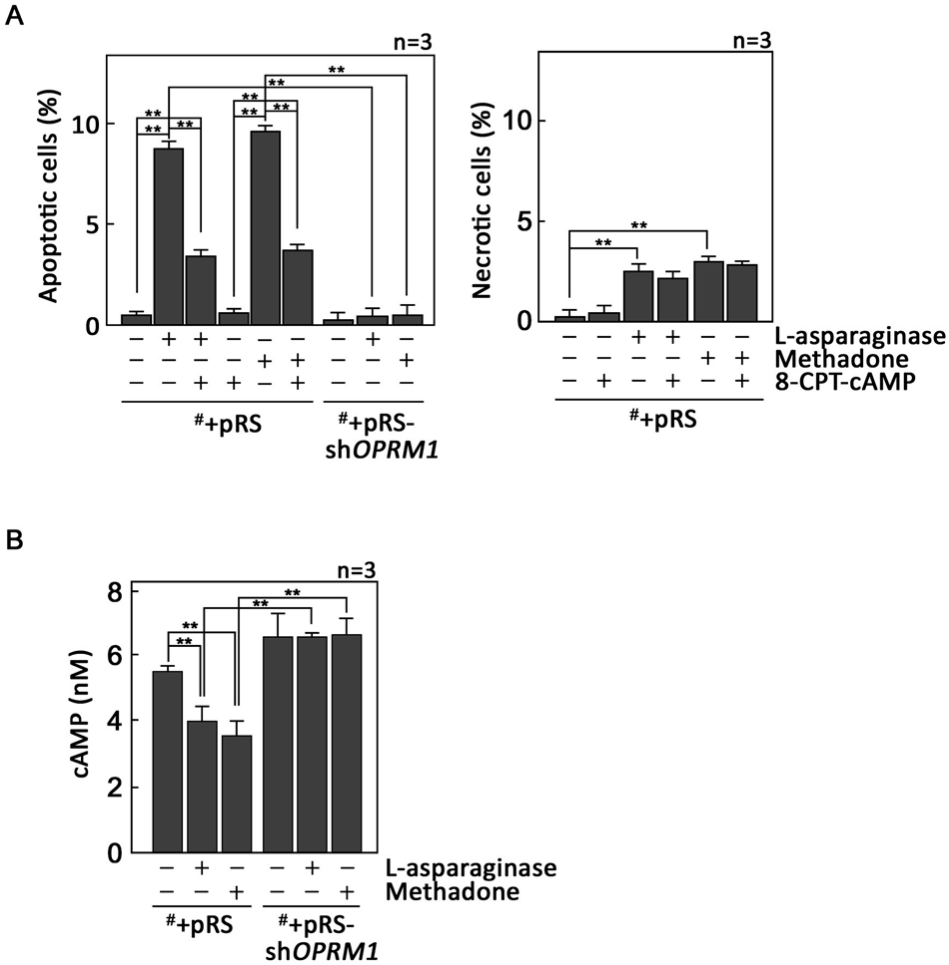
l-asparaginase-induced apoptosis is accompanied by a decrease in [cAMP]_i_. ^#^+pRS and ^#^+pRS-sh*OPRM1* cells pre-treated (or not pre-treated) with 50 nM 8-CPT-cAMP (a cAMP analogue) for 30 min then treated or untreated with 50 mIU/ml l-asparaginase or 0.5 μg/ml methadone (positive control) for 12 h were double-stained with PI- and FITC-labeled Annexin V then subjected to flow cytometry to measure early apoptotic (**A**, left panel) and necrotic (**A**, right panel) cell populations, and [cAMP]_i_ (**B**). Values are means ± SEM from three independent experiments. **p < 0.05.

To characterize the molecular mechanism through which l-asparaginase induces OPRM1-mediated aLL cell apoptosis, we used POETIC2 aLL cells (^#^) originating from a 14-year-old male patient with pre-B aLL [3]. These cells exhibit uninterrupted growth and survival in vitro. As a model system, these cells were infected with a retrovirus carrying either an empty pRS vector (^#^+pRS) or a pRS-sh*OPRM1* (TTGCACTGATGCCTTGGCG; ^#^+pRS-sh*OPRM1*) [3] to stably deplete OPRM1. As shown in Fig. 1A, ^#^+pRS cells express OPRM1 that is significantly reduced in ^#^+pRS-sh*OPRM1* cells. As expected, ^#^+pRS cells exhibit sensitivity to l-asparaginase, while OPRM1-depleted ^#^+pRS-sh*OPRM1* cells are resistant to l-asparaginase (Fig. 1B). Pre-treatment with a caspase inhibitor, Ac-DEVD-CHO, reverses l-asparaginase-induced apoptosis in ^#^+pRS cells (Fig. 1C), indicating that decreased cell viability following l-asparaginase treatment is due to caspase-mediated apoptotic cell death. In glioblastoma cells, d,l-methadone, an opioid receptor agonist, stimulates OPRM1 and instigates inhibitory G_i_ proteins, which inhibit adenylyl cyclase activity, lowering [cAMP]_i_ [5, 11]. We then tested whether OPRM1-mediated l-asparaginase-induced aLL cell apoptosis is accompanied by a decrease in [cAMP]_i_. To do so, cells treated with l-asparaginase for 12 h were analyzed for both apoptosis and [cAMP]_i_ by double-staining with PI- and FITC-labeled Annexin V followed by flow cytometry and using the cyclic-AMP XP® assay kit, respectively. As shown in Fig. 2 and Supplementary Fig. 1, ^#^+pRS cells treated with l-asparaginase show a significant (*p* < 0.05) increase in apoptosis (Fig. 2A, left panel; Supplementary Fig. 1) and decrease in [cAMP]_i_ (Fig. 2B). Conversely, ^#^+pRS-sh*OPRM1* cells treated with l-asparaginase show no significant apoptosis or change in [cAMP]_i_. These findings, which are consistent with those observed in cells treated with methadone (positive control), indicate that l-asparaginase-induced OPRM1-mediated aLL cell apoptosis involves a decline in [cAMP]_i_. This view is supported by our finding that pre-treatment with the lipophilic and cell-permeable cAMP analog, 8-CPT-cAMP, inhibits l-asparaginase-induced apoptosis in ^#^+pRS cells (Fig. 2A, left panel; and Supplementary Fig. 1). Apparently, l-asparaginase induces aLL cell apoptosis by stimulating the OPRM1 pathway to downregulate [cAMP]_i_. While l-asparaginase also induces necrosis, albeit to a lesser degree, in ^#^+pRS cells, pre-treatment with 8-CPT-cAMP does not affect the extent of necrosis (Fig. 2A, right panel), indicating that l-asparaginase-induced necrosis is not linked to the OPRM1-cAMP pathway.

### l-asparaginase upregulates pro-apoptotic BAX and BAK, but downregulates BAD S_118_ phosphorylation and anti-apoptotic BCL-2

**Fig. 3:**
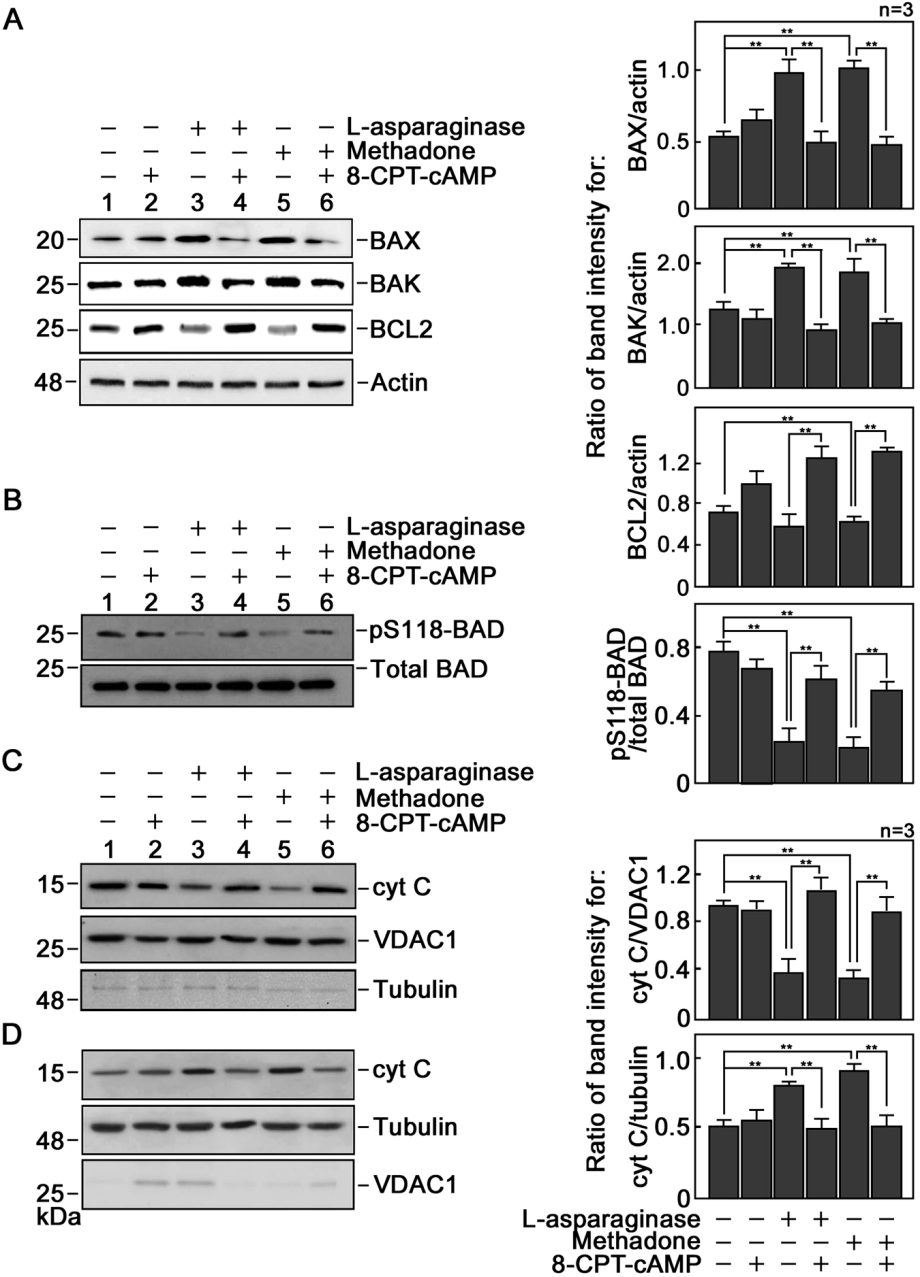
l-asparaginase causes downregulation of anti-apoptotic BCL-2 and BAD S_118_ phosphorylation, and upregulation of pro-apoptotic BAX and BAK, causing cyt C release from the mitochondria to the cytosol. Lysates of ^#^+pRS cells pre-treated (or not pre-treated) with 50 nM 8-CPT-cAMP for 30 min then treated or untreated with 50 mIU/ml l-asparaginase or 0.5 μg/ml methadone for 12 h were analyzed by immunoblotting for BCL-2, BAX, BAK and actin (**A**) and phosphoSer118-BAD (pS_118_–BAD) and BAD (**B**). Representative blots from one of three independent experiments (*n* = 3) showing similar results are shown. Graphs on the right panel indicate ratios of BCL-2, BAX or BAK levels vs actin levels, and pS_118_–BAD levels vs total BAD levels as measured by densitometric analysis of representative blots shown on the left panel. Densitometry was performed using the NIH ImageJ 1.61 software with the actin and total BAD values normalized to 1.0. Calculated ratios are means ± SD of the three independent experiments. **p < 0.05. ^#^+pRS cells pre-treated (or not pre-treated) with 50 nM 8-CPT-cAMP for 30 min then treated or untreated with 50 mIU l-asparaginase or 0.5 μg/ml methadone for 12 h were subjected to subcellular fractionation as described in “Materials and Methods”. The cytosolic (**C**) and mitochondrial (**D**) fractions were analyzed by immunoblotting for cyt C, tubulin (cytosolic marker) and VDAC-1 (mitochondrial marker). Representative blots from one of three independent experiments (*n* = 3) showing similar results are shown. Graphs on the right panel indicate ratios of cytosolic cyt C levels vs tubulin levels and mitochondrial cyt C levels vs VDAC levels as measured by densitometric analysis of representative blots shown on the left panel. The tubulin and VDAC-1 values were normalized to 1.0. Calculated ratios are means ± SD of the three independent experiments. **p < 0.05.

Interactions between the pro-apoptotic and anti-apoptotic BCL-2 family of proteins that regulate MOMP are influenced by the relative abundance of these proteins [6]. Thus, we examined the levels of the pro-apoptotic BAX and BAK and anti-apoptotic BCL-2 in ^#^+pRS cells treated with l-asparaginase. Western blot analysis shows an increase in pro-apoptotic BAX and BAK levels, but a decrease in anti-apoptotic BCL-2 level in cells treated with l-asparaginase compared to cells untreated with l-asparaginase (Fig. 3A). Methadone was used as a positive control. Since [cAMP]_i_-dependent PKA phosphorylation of BAD at S_118_ promotes anti-apoptotic activity of BCL-2 [7], we also examined BAD S_118_ phosphorylation in ^#^+pRS cells pre-treated (or not pre-treated) with 8-CPT-cAMP then treated with l-asparaginase. As shown in Fig. 3B, l-asparaginase treatment reduces BAD S_118_ phosphorylation in ^#^+pRS cells. Pre-treatment with 8-CPT-cAMP reverses this l-asparaginase effect on BAD S_118_ phosphorylation. Similarly, 8-CPT-cAMP reverses l-asparaginase effects on BAX, BAK and BCL-2 levels (Fig. 3A). These findings suggest that l-asparaginase stimulation of OPRM1 and subsequent decline in [cAMP]_i_ influence the expression or phosphorylation of the BCL-2 apoptotic and anti-apoptotic proteins, specifically upregulating pro-apoptotic BAX and BAK levels, and downregulating BAD S_118_ phosphorylation and anti-apoptotic BCL-2 level.

### l-asparaginase induces cyt C release from the mitochondria to the cytosol, resulting in caspase activation

**Fig. 4:**
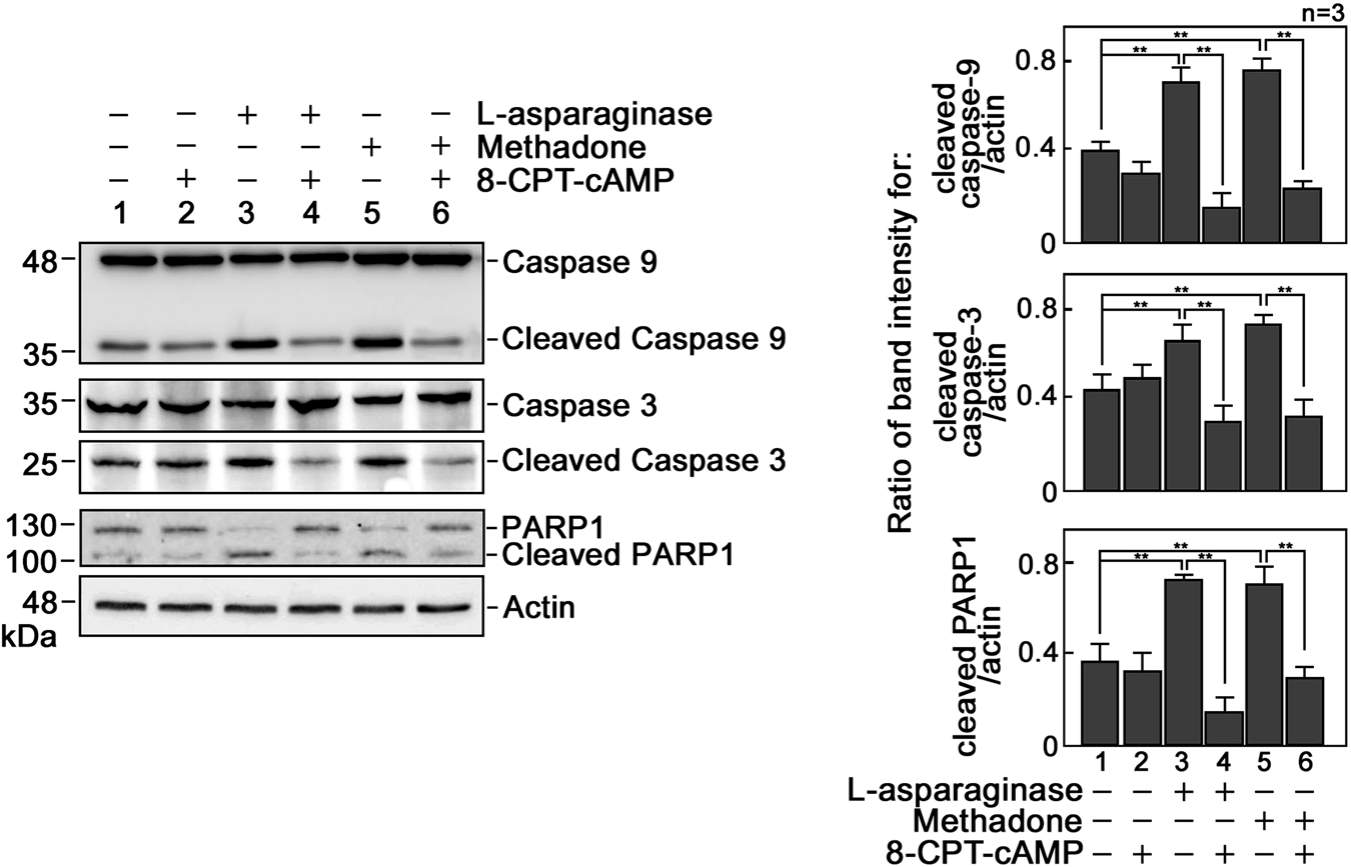
l-asparaginase causes caspase-3 and -9 activation. Lysates of ^#^+pRS cells pre-treated (or not pre-treated) with 8-CPT-cAMP for 30 min then treated or untreated with 50 mIU/ml l-asparaginase or 0.5 μg/ml methadone for 12 h were analyzed by immunoblotting for caspase-3 and -9, and PARP1. Representative blots from one of three independent experiments (*n* = 3) showing similar results are shown. Graphs on the right panel indicate ratios of cleaved caspase-3 and -9, and cleaved PARP1 vs actin levels as measured by densitometric analysis of representative blots shown on the left panel. The actin values were normalized to 1.0. Calculated ratios are means ± SD of the three independent experiments. **p < 0.05.

Since increased pro-apoptotic BAX and BAK levels, and decreased BAD S_118_ phosphorylation cause the formation of BAX/BAK oligomer pores that lead to MOMP [6], we next examined whether l-asparaginase causes cyt C release from the mitochondria. To do so, cells pre-treated (or not pre-treated) with 8-CPT-cAMP then treated with l-asparaginase were subjected to subcellular fractionation to isolate the mitochondrial and cytosolic fractions. Purity of these fractions was assessed based on the enrichment of VDAC (a mitochondrial protein) and lack of tubulin (a cytosolic marker) in the mitochondrial fraction (Fig. 3C), and vice versa in the cytosolic fraction (Fig. 3D). As shown in Fig. 3C, mitochondria from cells treated with l-asparaginase show decreased level of cyt C compared to those from control cells untreated with l-asparaginase (lanes 1 and 2). Conversely, the cytosolic fraction (Fig. 3D) from cells treated with l-asparaginase shows an increased level of cyt C compared to those from control cells untreated with l-asparaginase (lanes 1 and 2). Pre-treatment with 8-CPT-cAMP reverses the effect of l-asparaginase. These findings, which are consistent with those observed in cells treated with methadone (positive control) in the presence or absence of 8-CPT-cAMP, support the view that reduced [cAMP]_i_ triggers cyt C release from the mitochondria. Since cyt C release from the mitochondria is known to induce caspase activation and caspase-3-mediated PARP1 cleavage, we examined whether l-asparaginase-induced cyt C release also results in these caspase activation cascades. As shown in Fig. 4, l-asparaginase treatment causes activation of caspase-9 and -3 and subsequent PARP1 cleavage, which were also reversed by pre-treatment with 8-CPT-cAMP, further suggesting that reduced [cAMP]_i_ activates the caspase-PARP1 pathway. Methadone was again used as a positive control for this analysis.

### H89, a PKA inhibitor, promotes death of l-asparaginase-resistant aLL cells depleted of OPRM1

**Fig. 5:**
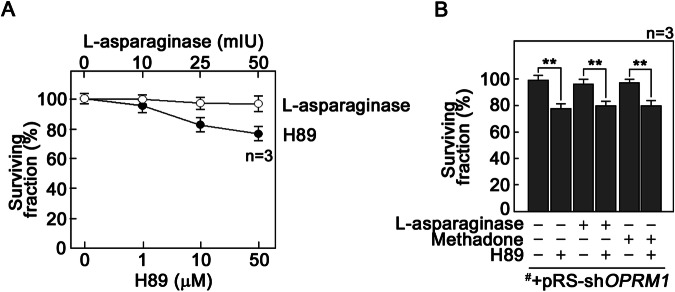
Inhibition of PKA kills l-asparaginase-resistant ^#^+pRS-sh*OPRM1* cells. **A** ^#^+pRS-sh*OPRM1* cells were treated with increasing concentrations (1, 10 and 50 μM) of the PKA inhibitor, H89, for 24 h, and cell viability was quantified using Alamar blue assay. Values are means ± SEM from three independent experiments. **B** Cells pre-incubated (or not pre-incubated) with 50 μM H89 were treated (or untreated) 1 h later with 50 mIU l-asparaginase or 0.5 μg/ml methadone for 24 h then analyzed for cell viability using Alamar blue assay. Values are means ± SEM of three independent experiments. **p < 0.05.

Altogether, our observations above indicate that [cAMP]_i_ as a key component of the OPRM1-mediated l-asparaginase-induced apoptotic pathway. Since reduced [cAMP]_i_ can lead to decreased PKA activation, which promotes apoptosis, we sought to determine whether PKA can be targeted to kill l-asparaginase-resistant aLL cells deficient in OPRM1. To do so, we examined the effect of H89, an isoquinoline sulfonamide and inhibitor of PKA, on the response of OPRM1-knockdown ^#^+pRS-sh*OPRM1* cells to l-asparaginase. As shown in Fig. 5A, ^#^+pRS-sh*OPRM1* cells are resistant to treatment with 10–50 mIU l-asparaginase (○). However, H89 kills ^#^+pRS-sh*OPRM1* cells in a dose-dependent manner (Fig. 5A, ●) with 50 μM H89 killing ~20% of l-asparaginase-resistant ^#^+pRS-sh*OPRM1* cells at 24 h. Figure 5B shows that as with l-asparaginase-resistant ^#^+pRS-sh*OPRM1* cells, around 20% of methadone-resistant ^#^+pRS-shOPRM1 cells can be killed by treatment with 50 μM H89.

### PKA inhibition by H89 or 14–22 amide promotes death of patient aLL cells that exhibit l-asparaginase resistance linked to reduced OPRM1 expression

**Fig. 6:**
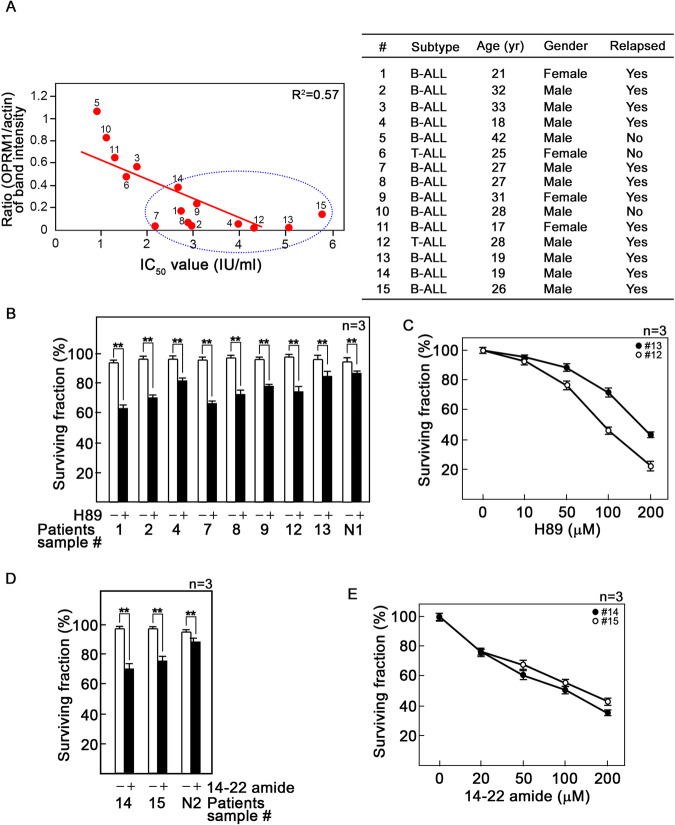
Inhibition of PKA kills l-asparaginase-resistant patient aLL cells with reduced OPRM1 levels. **A** Reduced OPRM1 levels in patient aLL cells correlate with increased resistance to l-asparaginase. The panel (*n* = 15) of primary aLL cell samples isolated from patient peripheral blood were treated with different concentrations of l-asparaginase. IC50 values were calculated as described previously [3, 14] using Graphpad Prism 7 after plotting l-asparaginase dose-dependent survival of aLL cells as measured by Alamar blue assay. Ratios of the OPRM1 vs actin levels in patient samples (*n* = 15) were determined following densitometry analysis of immunoblots using the NIH ImageJ 1.61 software. Numbers next to the graph symbol () correspond to the patient designation number (#) indicated in the table at the right panel, which also shows other patient information. Inhibition of PKA by H89 (**B**, **C**) or 14–22 amide (**D**, **E**) kills l-asparaginase-resistant patient aLL cells. Primary aLL cells that express reduced OPRM1 levels and exhibit resistance to l-asparaginase (Fig. 6A, left panel, enclosed in dotted circle) were treated with 50 μM H89 (**B**) or 20 μM 14–22 amide (**D**) for 24 h. Cell viability was assessed using Alamar blue assay. Values are means ± SEM of three independent experiments (*n* = 3). **p < 0.05. Dose-dependent killing of representative relapsed patient aLL cells by H89 (**C**, patients #12 and #13) and 14–22 amide (**E**, patients #14 and #15). Cells were treated with increasing concentrations of H89 (**C**) or 14–22 amide (**E**) for 24 h. Cell viability was assessed using Alamar blue assay. Values are means ± SEM of three independent experiments (*n* = 3).

Figure 6A, left panel, shows the OPRM1 expression levels and corresponding l-asparaginase IC50 values in leukemic cells isolated from 15 aLL patients. Analysis indicates that OPRM1 level is inversely proportional to l-asparaginase IC50: the lower the OPRM1 level, the higher the l-asparaginase IC50, indicating that aLL cells expressing reduced OPRM1 level show resistance to l-asparaginase. We note that of the 15 patient samples analyzed, 10 out of 12 leukemic cell samples from relapsed patients (Fig. 6A, right panel) have reduced OPRM1 levels and increased IC50 values compared to non-relapsed patients. To assess the clinical relevance of targeting PKA in aLL cells that exhibit l-asparaginase resistance due to reduced OPRM1 levels, we utilized the l-asparaginase-resistant and low OPRM1-expressing aLL cells from the ten patients indicated above (Fig. 6A, enclosed in dotted circle). As shown in Fig. 6B, H89 at 50 μM kills up to 40% of these cells (■). Under the same conditions, only ~7% of normal lymphocytes from healthy individuals is killed. Increasing concentrations of H89 cause a corresponding increase in killing of relapsed patient primary aLL cells, with 200 μM causing ~80% and ~57% cell death in patients #12 and #13, respectively (Fig. 6C). As shown in Fig. 6D, 20 μM of the PKA-specific inhibitor, 14–22 amide [12], kills up to 30% of the relapsed patients #14 and #15 primary aLL cells (■), while only ~6% of normal lymphocytes from healthy individuals is killed. As with H89, increasing concentrations of 14–22 amide cause a corresponding increase in killing of relapsed patient primary aLL cells, with 200 μM causing ~65% and ~57% cell death in patients #14 and #15, respectively (Fig. 6E).

## Discussion

In this study, we present the very first analysis of the OPRM1-mediated l-asparaginase-induced apoptosis in aLL cells. The use of OPRM1-expressing and -knockdown aLL cells infected with retrovirus carrying a pRS empty vector and pRS-sh*OPRM1*, respectively, as model systems allowed us to show that l-asparaginase-induced OPRM1-mediated aLL cell apoptosis is associated with decline in [cAMP]_i_. The lipophilic and cell-permeable cAMP analog, 8-CPT-cAMP, reverses this apoptotic process, indicating the critical involvement of cAMP in the apoptotic pathway. Our observations rule out a relevant role for OPRM1-cAMP in the modest level of necrosis induced by l-asparaginase.

Our finding that 8-CPT-cAMP also reverses the l-asparaginase-induced decline in S_118_–BAD phosphorylation in aLL cells indicates an important role for loss of cAMP-dependent PKA phosphorylation of BAD S_118_ in l-asparaginase-induced aLL cell apoptosis. While BAD is also phosphorylated at S_118_ by ribosomal S6 kinase 1 [8], and protein kinase C iota (PKC-iota) [13], the fact that 8-CPT-cAMP can bring the l-asparaginase-induced decline in S_118_–BAD phosphorylation back almost up to levels similar to those in control cells suggests that PKA is the major kinase involved in BAD S_118_ phosphorylation. Our finding also indicates that loss of PKA-mediated BAD S_118_ phosphorylation is crucial for OPRM1-mediated l-asparaginase-induced apoptosis in aLL cells.

**Fig. 7:**
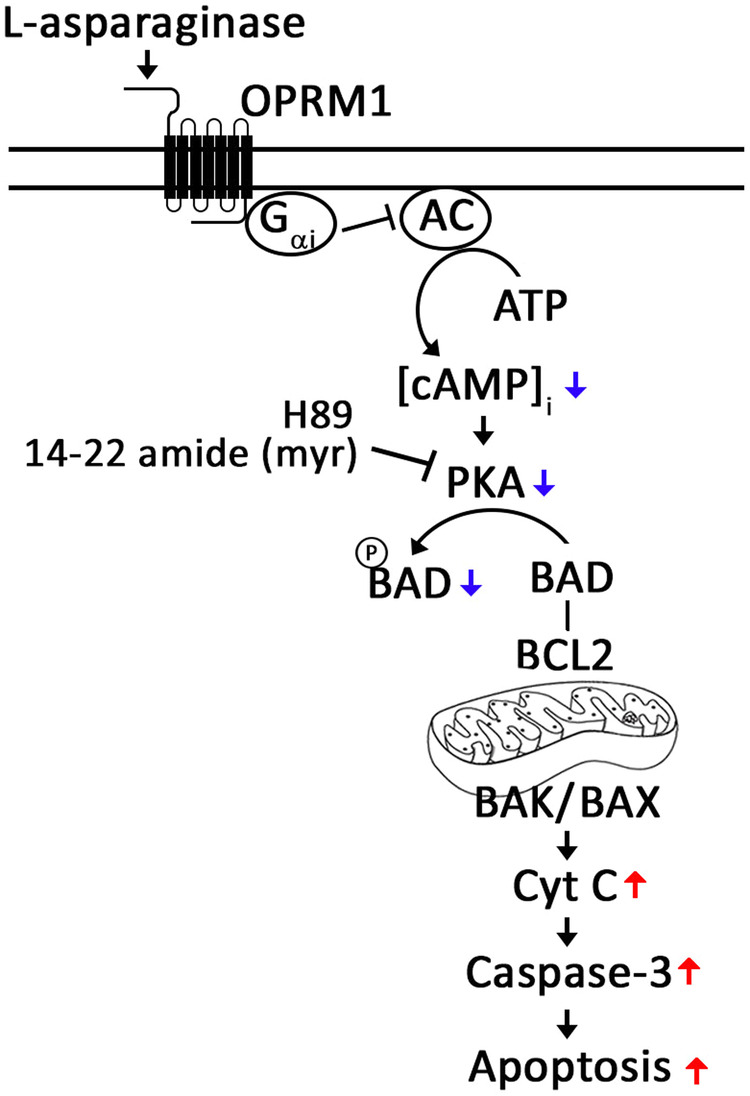
Proposed mechanism for l-asparaginase-induced OPRM1-mediated apoptosis in aLL cells. l-asparaginase causes an OPRM1-mediated decline in [cAMP]_i_ that is linked to reduced PKA-associated phosphorylation of BAD at S_118_, which inhibits the anti-apoptotic role of BCL-2, positively regulating MOMP and stimulating the release of mitochondrial intermembrane space proteins, such as cytochrome C, causing caspase activation and consequently, aLL cell apoptosis.

The relative abundance of the BCL-2 family of proteins such as the anti-apoptotic BCL-2 and the pro-apoptotic pore-formers, BAX/BAK, govern MOMP. Our findings that l-asparaginase causes an increase in BAX and BAK levels and a decrease in BCL-2 level in aLL cells, and that 8-CPT-cAMP can reverse these l-asparaginase effects indicate that these alterations in BAX, BAK and BCL-2 levels are further linked to l-asparaginase-induced OPRM1-mediated [cAMP]_i_ decline. Evidently, l-asparaginase induces aLL cell apoptosis via a cascade of events that include upregulation of pro-apoptotic BAX and BAK and downregulation of anti-apoptotic BCL-2, apoptosis-associated increase in cyt C release, caspase activation, and PARP1 cleavage that follows OPRM1-mediated downregulation of [cAMP]_i_ and reduced PKA-mediated BAD S_118_ phosphorylation (Fig. 7). This l-asparaginase-induced apoptotic pathway via OPRM1 is distinct from our previously characterized l-asparaginase-induced apoptotic pathway that is mediated by huntingtin-associated protein 1 (HAP1) [14]. HAP1 forms a ternary complex with huntingtin (Htt) and the intracellular Ca^2+^ channel IP3R, allowing l-asparaginase-induced IP3R-mediated ER Ca^2+^ release in aLL cells. HAP1 loss inhibits the formation of the HAP1–Htt–IP3R complex. The subsequent loss of ER Ca^2+^ release confers l-asparaginase resistance. On the other hand, presence of HAP1 allows the ternary complex formation and ER Ca^2+^ release, perturbing intracellular Ca^2+^ homeostasis, and activation of calpain-1-Bid-cyt C-caspases apoptotic pathway [14]. Since PKA phosphorylates PLCβ3 at serine 1105 and downregulates its activity and thus ER Ca^2+^ release [15], the IP3-induced IP3R [14]- and the OPRM1-mediated pathways [3, 5] may be linked through PKA. This possibility is currently under investigation.

It is notable that a potent PKA inhibitor, H89, can promote death of l-asparaginase-resistant aLL cells depleted of OPRM1, and patient aLL cells that exhibit l-asparaginase resistance due to reduced OPRM1 expression. Although H89 also targets serum and glucocorticoid-regulated kinase, rho-associated protein kinase 2,5′-AMP-activated protein kinase, checkpoint kinase 1, ribosomal protein S6 kinases b1 and 2 (also known as MAPKAP-K1b), mitogen- and stress-activated protein kinase-1, and protein kinase B-α, these are not involved in pro-apoptotic BAD S_118_ phosphorylation. Specific PKA inhibition using 14–22 amide causes similar effects as H89. Our observations that H89 and 14–22 amide kill up to ~80% and ~65%, respectively, of l-asparaginase-resistant patient aLL cells, and only ~7 and 6% of normal lymphocytes, respectively, from healthy individuals, while having a variable killing effect among patient aLL cells, indicate that aLL cell killing is not due to toxicity. Thus, we demonstrate for the first time that PKA can be targeted to kill l-asparaginase-resistant aLL cells lacking OPRM1, and that H89 or 14–22 amide may be utilized to inhibit PKA and kill patient aLL cells.

Although the mechanism of OPRM1 activation by methadone, an μ-opioid receptor agonist, has been previously characterized [16], there are no reports on how l-asparaginase activates OPRM1. l-asparaginase is known to hydrolyze asparagine into aspartate and NH_3_ and shown to have β-aspartyl peptidase activity [17–20]. Potentially, l-asparaginase utilizes its enzymatic activity and/or interacts with OPRM1 to activate the OPRM1-mediated apoptotic pathway. While this remains to be explored, our current studies reveal the mechanisms by which OPRM1 activation by l-asparaginase induces aLL cell death. Most importantly, we propose that inhibition of PKA activity by H89 or 14–22 amide may serve as an effective therapy for aLL patients with leukemic cells deficient in OPRM1.

## Materials and methods

### Materials

Opti-MEM (31985062), penicillin–streptomycin (15140122) and pSer118-BAD (PA5-12550) were from ThermoFisher Scientific (Burlington, ON, Canada). FBS (89510-186) was from Avantor (Radnor, PA, USA). l-asparaginase was from Abcam (ON, Canada). Antibodies to PARP1 (F-2), caspase-3 (E-8), caspase-9 (3C22), BAD (C-7), actin (C-2), cytochrome C (cyt C, H-104), VDAC-1 (B-6) and tubulin (D-10) were from Santa Cruz Biotechnology (TX, USA). Antibody to cleaved caspase-3 (D1-75) was from Cell Signaling (ON, Canada). The cAMP analog, 8-(4-chlorophenylthio)adenosine 3′,5′-cyclic monophosphate (8-CPT-cAMP), Ac-DEVD-CHO and the 14–22 amide PKA inhibitor were from Sigma Aldrich (MO, USA). The PKA inhibitor, N-[2-(p-Bromocinnamylamino)ethyl]-5-isoquinolinesulfonamide 2HCl (H89) was from Santa Cruz Biotechnology (Dallas, TX, USA).

### Cell culture

POETIC2 cells, a gift from Dr. Aru Narendran, University of Calgary, were derived from a 14-year-old pre-B aLL patient [3]. Cells were cultured in opti-MEM supplemented with 10% FBS and 100 units/ml each of penicillin and streptomycin at 37 °C and CO_2_ level of 5%. Cells were tested for mycoplasma contamination.

### Patient leukemic cell isolation

Patient leukemic cells were prepared from peripheral blood samples of aLL patients at the Second Affiliated Hospital (Harbin, China), based on the protocol approved by the Ethics Committee at Harbin Medical University (HMUIRB20150023; where informed consent was obtained from all subjects) and the Health Research Ethics Board of Alberta Cancer Committee (HREBA.CC-17-0108_REN7).

### Determination of cell viability

Cells (1 × 10^4^ cells/well in a 96-well plate) were treated with the indicated concentrations of l-asparaginase and/or H89 or 14–22 amide for 1 day. Cell viability was quantified using Alamar Blue assay (Life Technologies, USA) as per the manufacturer’s recommendations.

### Measurement of [cAMP]_i_

Cells (1 × 10^6^ cells/well in a 6-well plate) treated or untreated with 50 mIU/ml l-asparaginase or 0.5 μg/ml methadone for 30 min were subjected to [cAMP]_i_ measurement using the cyclic-AMP XP® assay kit (Cell Signaling, USA), and according to the manufacturer’s protocol. Briefly, 50 μl of cell lysates were added to each well in a 96-well cyclic-AMP XP® assay kit plate. cAMP present in the samples competes with a fixed amount of HRP-linked cAMP for binding to anti-cAMP XP® rabbit monoclonal antibody immobilized on the wells. After washing off unbound HRP-linked cAMP, 50 μl of the HRP substrate, TMB, was added. After 30 min, the reaction was stopped using 50 μl of sulfuric acid. The concentration of bound cAMP from the samples was then measured at 450 nm. [cAMP]_i_ in cell lysates was calculated based on a cAMP standard curve.

### Measurement of apoptotic and necrotic cell populations

Cells (0.5 × 10^6^) seeded in 6-cm dishes were pre-treated (or not pre-treated) with 50 nM 8-CPT-cAMP for 30 min, then treated (or untreated) with 50 mIU/ml of l-asparaginase or 0.5 μg/ml of methadone for 12 h. After harvesting, cells were washed twice with 1× PBS by centrifugation at 1000 rpm for 5 min, then resuspended in 100 μl of staining buffer (from the Annexin V-FITC staining Kit, Invitrogen) and stained by adding 2 μl of Annexin V-FITC and 2 μl of propidium iodide (PI). Stained cells were subsequently analyzed by flow cytometry (Attune NxT flow cytometer, ThermoFisher Scientific, USA).

### Western blot analysis

Cell lysates separated by 12.5% SDS–PAGE were transferred to nitrocellulose membranes (Pall Laboratory, ON), which were then immunostained for BAD, pSer118-BAD, cyt C, VDAC-1, tubulin, caspase-3, cleaved caspase-3, caspase-9, PARP1 or actin. Membranes were incubated with antibodies (diluted to 1:1000) overnight at 4 °C. After washing three times in tris-buffered saline containing 0.1% triton X-100 (TBS-T), membranes were incubated with an HRP-conjugated secondary antibody (1:10,000) in TBS-T for 1 h. Immunoreactive protein bands were visualized using a ChemiDoc Imager (Bio-Rad) under the optimal exposure setup. No enhancements were performed. Relative levels of intensity of protein bands of interest were measured by densitometry analysis using the National Institutes of Health (NIH) ImageJ 1.61 software.

### Measurement of cytochrome C (cyt C) level

Subcellular fractionation was performed as described previously [21]. Briefly, cells harvested by centrifugation at 370 × *g* for 10 min were washed twice with 10 packed cell volumes of NKM buffer (1 mM Tris-HCl, pH 7.4, 0.13 M NaCl, 5 mM KCl and 7.5 mM MgCl_2_), resuspended in six packed cell volumes of homogenization buffer (10 mM Tris-HCl, pH 6.7, 10 mM KCl, 0.15 mM MgCl_2_, 1 mM PMSF and 1 mM DTT), and then homogenized using a glass homogenizer, resuspended in 2 M sucrose solution and centrifuged at 1200 × *g* for 5 min. The supernatant was further centrifuged at 7000 × *g* for 10 min and the resulting supernatant was called as cytosolic fraction. Pellets, which contain mitochondria, were resuspended with mitochondrial suspension buffer (10 mM Tris-HCl, pH 6.7, 0.15 mM MgCl_2_, 0.25 M sucrose, 1 mM PMSF and 1 mM DTT) and centrifuged at 10,000 × *g* for 5 min. Pellets were called as mitochondrial fraction. Isolated cytosolic and mitochondrial fractions were subjected to immunoblotting as described above.

### Statistical analysis

Results were presented as the means ± SD or SEM. Data were statistically analyzed using Student’s *t*-test (paired, two-tailed). Statistical significance was set at *p* < 0.05.

### Supplementary information

supplementary Figure legend

supplementary Figure 1

Uncropped western blots

## Data Availability

All data generated or analyzed during this study are included in this published article and its Supplementary Information files/uncropped data files.
