# Supplementary material for: PKA inhibition kills l-asparaginase-resistant leukemic cells from relapsed acute lymphoblastic leukemia patients
Source: Cell Death Discov. 2024 May 27;10:257. doi: 10.1038/s41420-024-02028-w (PMC11130271; doi:10.1038/s41420-024-02028-w)
Supplement: Supplementary file 1 — supplementary Figure legend [file 41420_2024_2028_MOESM1_ESM.docx]

**Supplementary Figure Legend**

**Supplementary Figure 1.** Representative flow cytometry data for Figure 2A. ^#^+pRS and ^#^+pRS-sh*OPRM1* cells pre-treated (or not pre-treated) with 50 nM 8-CPT-cAMP (a cAMP analogue) for 30 min then treated or untreated with 50 mIU/ml L-asparaginase or 0.5 μg/ml methadone (positive control) for 12 hrs were double-stained with PI- and FITC-labeled Annexin V then subjected to flow cytometry. Representative data from one of three independent experiments (n=3) showing similar results are shown.
