# Supplementary figures and images for: PKA inhibition kills l-asparaginase-resistant leukemic cells from relapsed acute lymphoblastic leukemia patients
Source: Cell Death Discov. 2024 May 27;10:257. doi: 10.1038/s41420-024-02028-w (PMC11130271; doi:10.1038/s41420-024-02028-w)

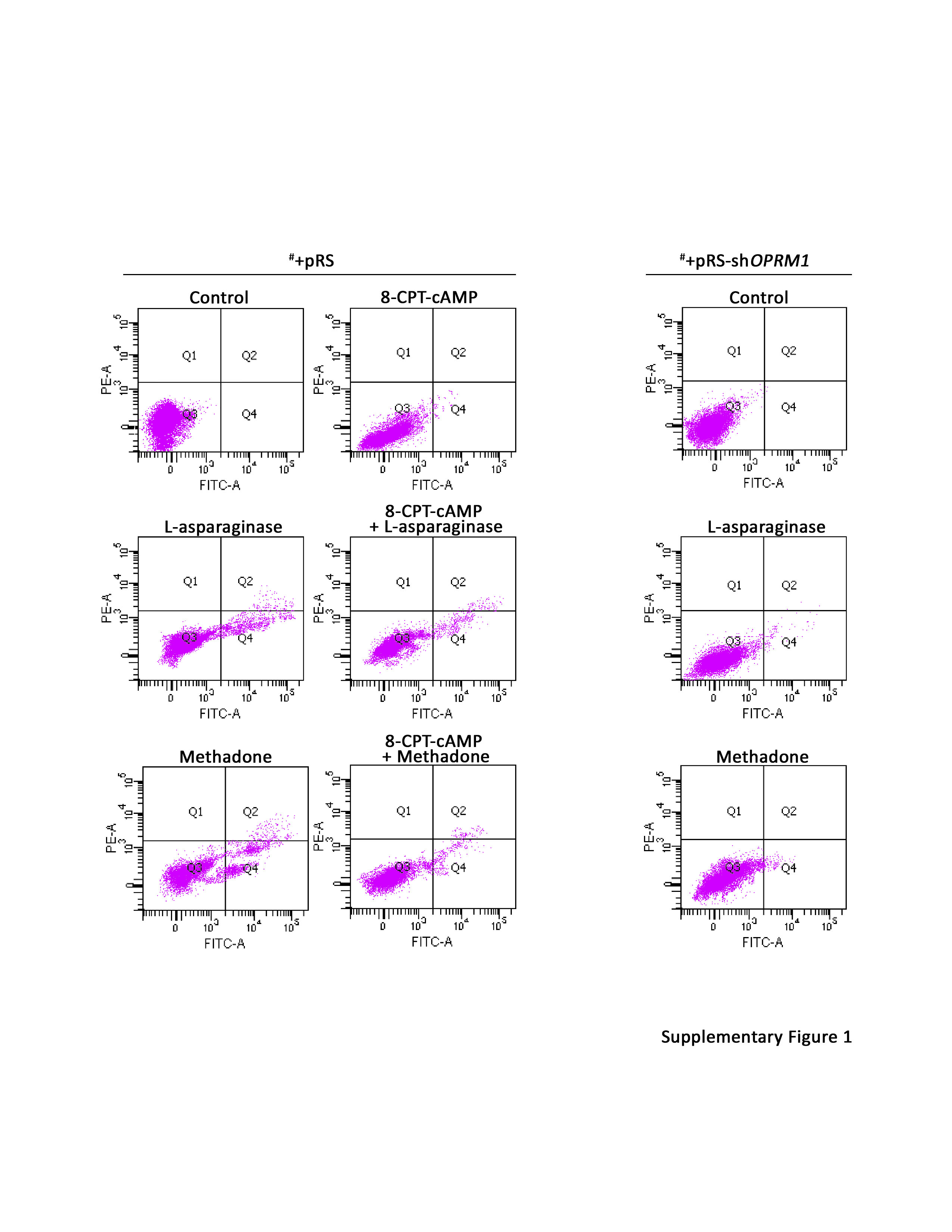

Supplement: Supplementary file 2 — supplementary Figure 1 [file 41420_2024_2028_MOESM2_ESM.jpg]
