# Supplementary material for: PKA inhibition kills l-asparaginase-resistant leukemic cells from relapsed acute lymphoblastic leukemia patients
Source: Cell Death Discov. 2024 May 27;10:257. doi: 10.1038/s41420-024-02028-w (PMC11130271; doi:10.1038/s41420-024-02028-w)
Supplement: Supplementary file 3 — Uncropped western blots [file 41420_2024_2028_MOESM3_ESM.pdf]

Note: some of the blots were cut to allow immunoblotting with different antibodies without reblotting the whole membrane.

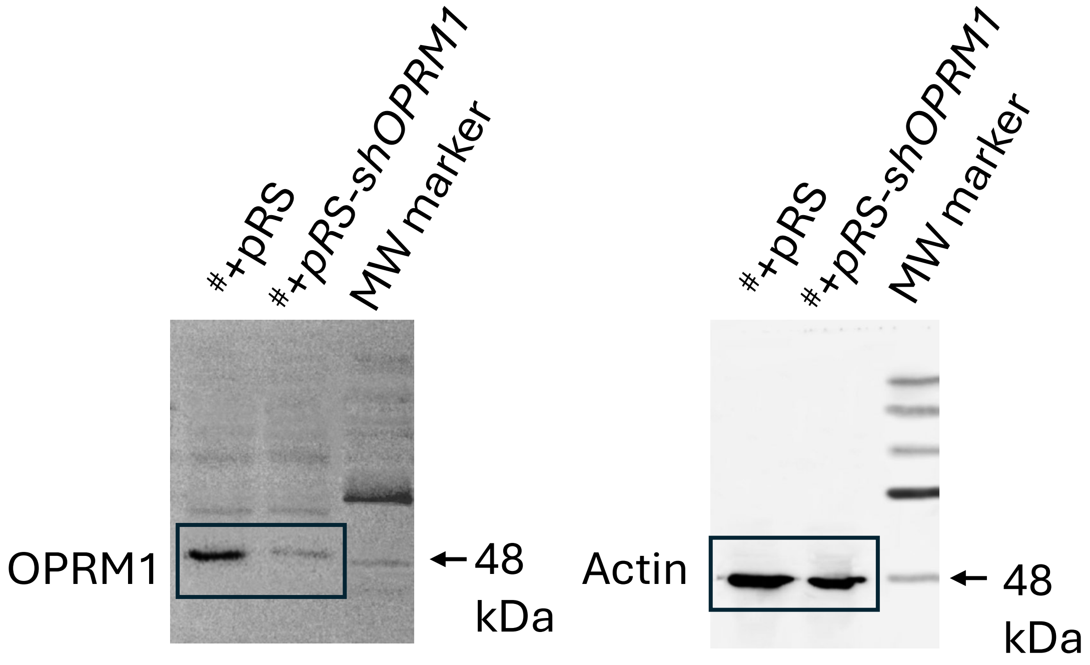


Figure 1A

**Figure 3A**

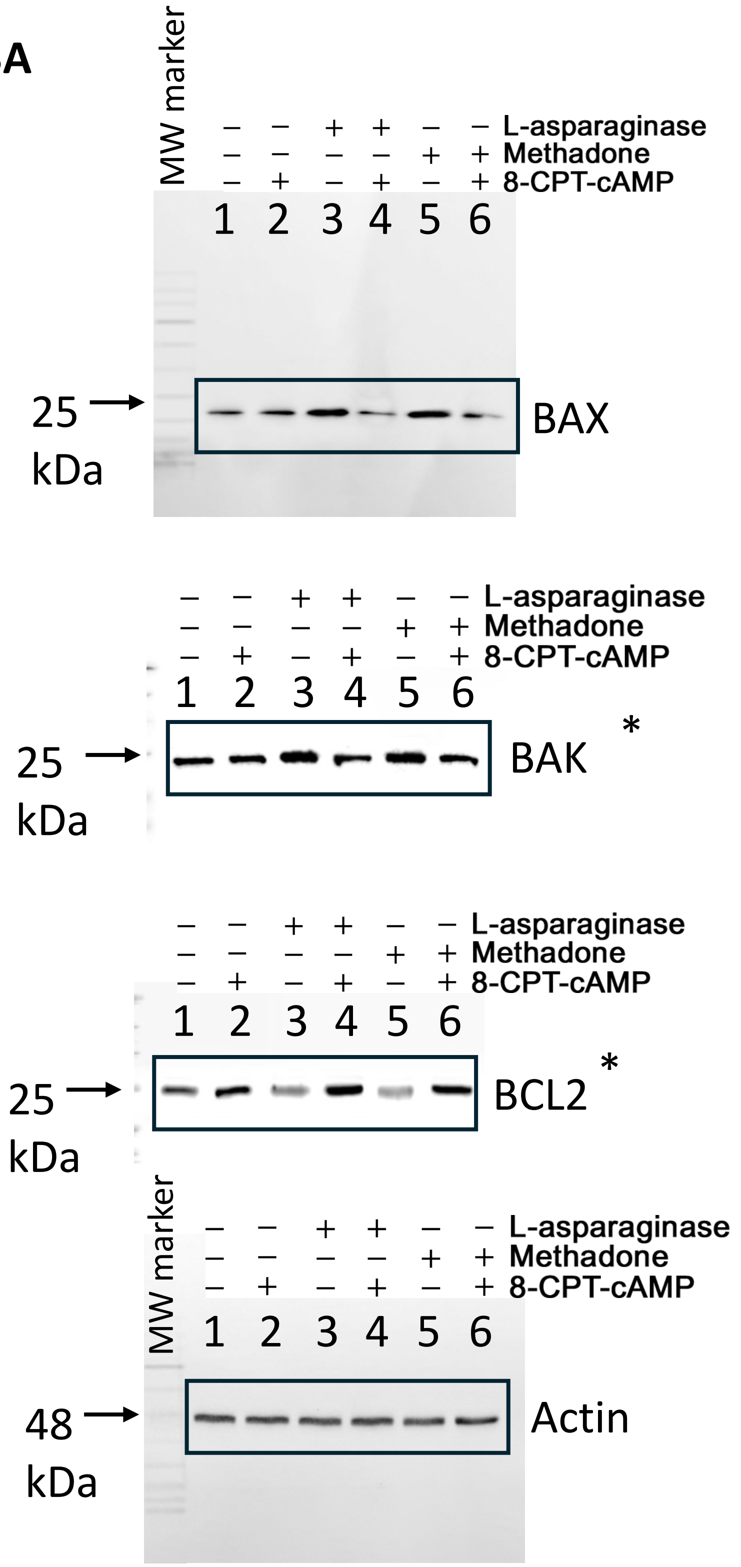


*Note: some of the blots were cut to allow immunoblotting with different antibodies without reblotting the whole membrane.

Figure 3

**Figure 3B**

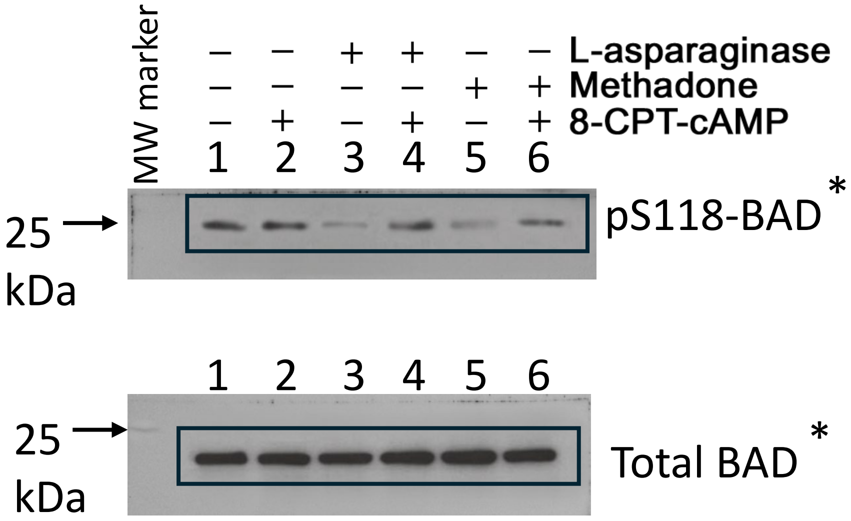


*Note: some of the blots were cut to allow immunoblotting with different antibodies without reblotting the whole membrane.

## Figure 3C

## Figure 3D

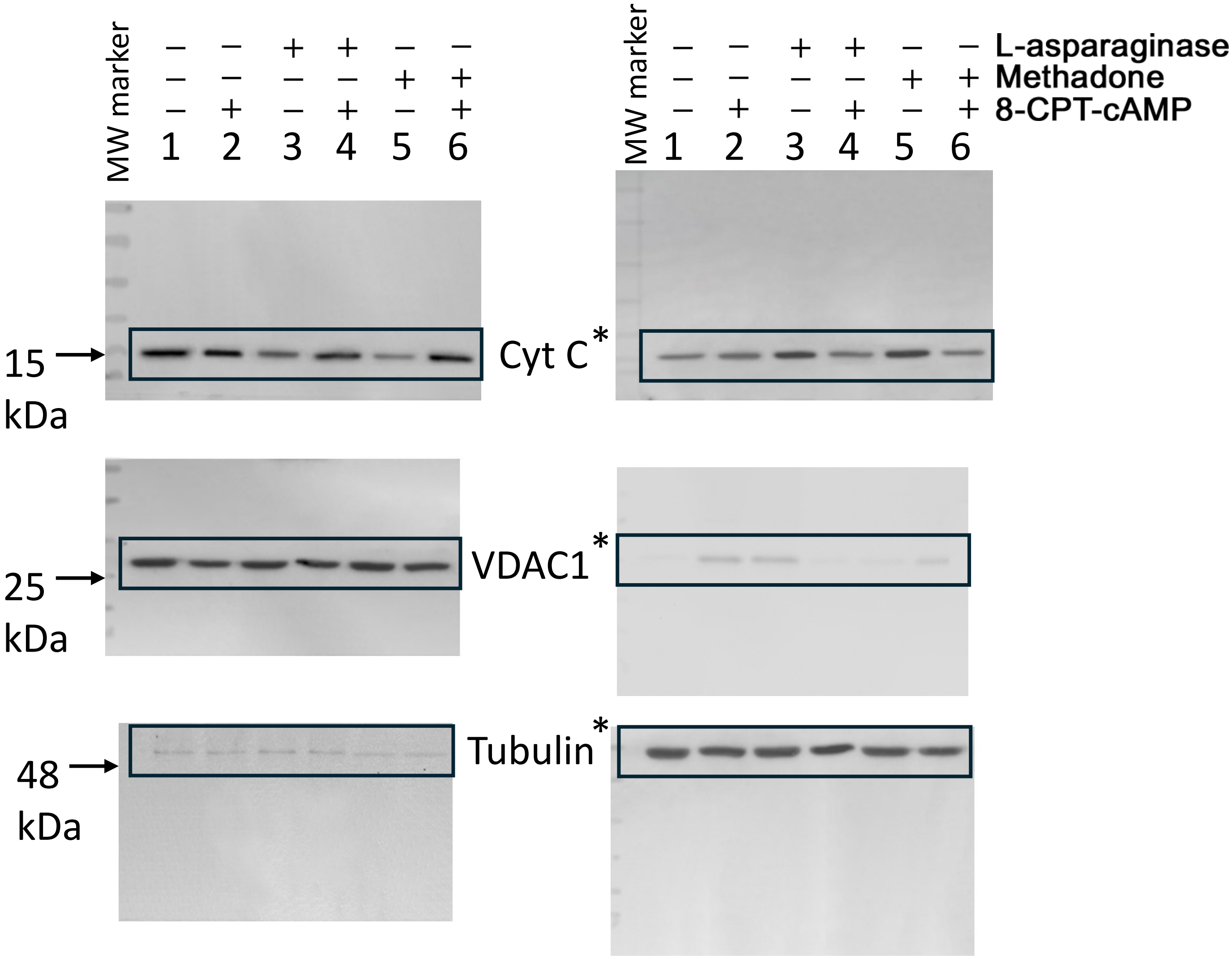


*Note: some of the blots were cut to allow immunoblotting with different antibodies without reblotting the whole membrane.

Figure 3

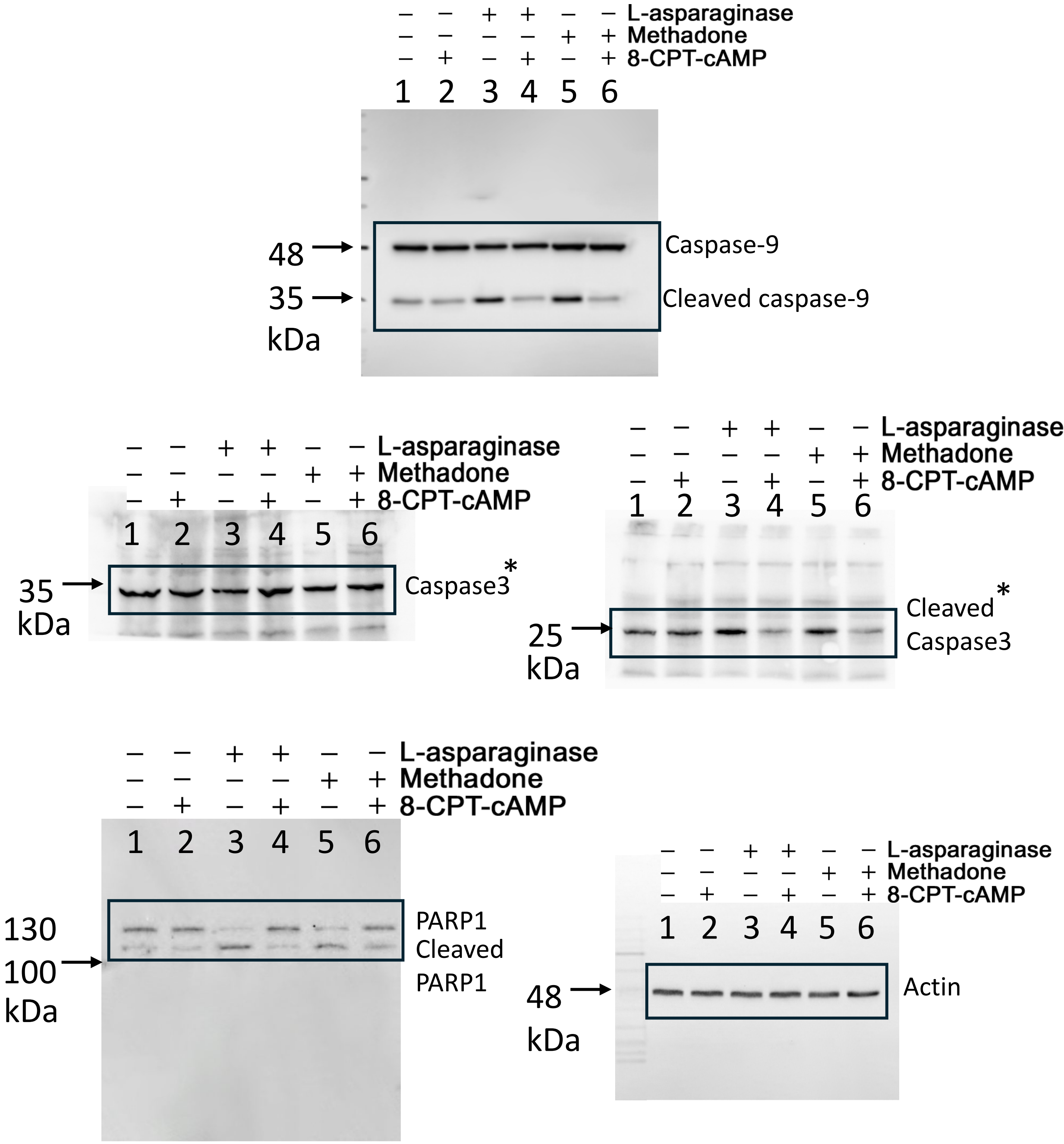


*Note: some of the blots were cut to allow immunoblotting with different antibodies without reblotting the whole membrane.

Figure 4
